# Non-Expresser of PR-Genes 1 Positively Regulates Abscisic Acid Signaling in *Arabidopsis* *thaliana*

**DOI:** 10.3390/plants11060815

**Published:** 2022-03-18

**Authors:** Irfan Ullah Khan, Akhtar Ali, Shah Zareen, Haris Ali Khan, Chae Jin Lim, Junghoon Park, Jose M. Pardo, Dae-Jin Yun

**Affiliations:** 1 Department of Biomedical Science & Engineering, Konkuk University, Seoul 05029, Korea; irfanmhmand@gmail.com (I.U.K.); gultkr@yahoo.com (A.A.); shahzareen75@gmail.com (S.Z.); hariskhangcu@gmail.com (H.A.K.); kirays2@nate.com (C.J.L.); p6259j@gmail.com (J.P.); 2 Institute of Glocal Disease Control, Konkuk University, Seoul 05029, Korea; 3 Institute of Plant Biochemistry and Photosynthesis, Consejo Superior de Investigaciones Cientificas, 41092 Seville, Spain; jose.pardo@csic.es

**Keywords:** ABA signaling, NPR1, HOS15, CULLIN4-DDB1, protein-protein interactions, protein degradation and stability

## Abstract

The plant hormone, abscisic acid (ABA), is not only important for promoting abiotic stress responses but also plays a versatile and crucial role in plant immunity. The pathogen infection-induced dynamic accumulation of ABA mediates the degradation of non-expresser of PR genes 1 (NPR1) through the CUL3^NPR3NPR4^ proteasome pathway. However, the functional significance of NPR1 degradation by other E3 ligases in response to ABA remains unclear. Here, we report that NPR1 is induced transcriptionally by ABA and that *npr1-1* mutation results in ABA insensitivity during seed germination and seedling growth. Mutants lacking *NPR1* downregulate the expression of ABA-responsive transcription factors ABA INSENSITIVE4 (ABI4) and ABA INSENSITIVE5 (ABI5), and that of their downstream targets *EM6*, *RAB18*, *RD26*, and *RD29B*. The *npr1-1* mutation also affects the transcriptional activity of WRKY18, which activates *WRKY60* in the presence of ABA. Furthermore, NPR1 directly interacts with and is degraded by HOS15, a substrate receptor for the DDB1-CUL4 ubiquitin E3 ligase complex. Collectively, our findings demonstrate that NPR1 acts as a positive regulator of ABA-responsive genes, whereas HOS15 promotes NPR1 degradation in a proteasome-dependent manner.

## 1. Introduction

Plants are constantly exposed to various biotic and abiotic stresses and have evolved sophisticated mechanisms to cope with harsh environmental conditions [1]. Understanding the mechanisms underlying such adaptations is critical for securing the yield of crop plants in agriculture [2]. Phytohormones such as abscisic acid (ABA), ethylene, salicylic acid (SA), and jasmonic acid are key regulators of plant responses to adverse environmental conditions. Among these, ABA is the most extensively involved in plant abiotic stress responses, including high salinity, drought, heat, and osmotic stress [3,4,5]. ABA regulates plant growth and developmental processes, including seed dormancy, seed germination, and stomatal movement. Recent studies have shown that the crosstalk of multiple signaling pathways is regulated by phytohormones either antagonistically or synergistically between biotic and abiotic interactions [5,6]. During vegetative growth, plant responses to various environmental stresses, including drought, salinity, low temperature, oxidative stress, mechanical wounding, and pathogen attack, can be divided into ABA-dependent and ABA-independent pathways [3,4,7,8,9,10,11,12]. Previous studies have shown that there are two main types of ABA-dependent pathways in plants. The first is mediated by the basic leucine zipper/ABA-responsive element (bZIP/ABRE) system [13]. These include ABA INSENSITIVE5 (ABI5)/ABF transcription factors (ABA-responsive element binding factors) that upregulate the expression of ABA-induced genes, such as *RD29A* and *RD29B*, under stress conditions [14,15,16]. ABA also prompts MYC/MYB transcription factors (MYC2 and MYB2) to induce drought responsive *RD22* expression in *Arabidopsis* [7,17]. Several downstream components important for ABA signaling have been characterized in the recent past. For instance, ABI1 and ABI2 are group A type-2C protein phosphatases that negatively regulate SNF1-related protein kinases 2 (SnRK2s), which phosphorylate and activate downstream AREB/ABF targets [18].

The transcriptional co-activator NPR1 regulates local and systemic acquired resistance (SAR) in response to SA. In *Arabidopsis thaliana*, cellular activities of NPR1 are regulated by intricate mechanisms. A defense-associated increase in SA levels induces *NPR1* transcriptional activation [19]. Next, SA induces cellular changes in the redox state that promote NPR1 monomerization and nuclear localization [20]. Moreover, NPR1 protein levels are constantly regulated by their proteasome-dependent turnover [21]. Furthermore, the direct binding of SA induces conformational changes in NPR1, leading to the release of the NPR1 transactivation domain from its autoinhibitory domain [22,23,24]. Finally, SA-induced NPR1 phosphorylation on Ser11 and Ser15 promotes NPR1 transcriptional coactivator activity, its recruitment to the CUL3 ^NPR3NPR4^-based E3-ligase complex, and subsequent protein turnover [21]. In line with this, phosphorylation of NPR1 by SnRK2.8 at S589 and T373 is required for its nuclear translocation [25]. In previous studies, two NPR1 paralogs, NPR3 and NPR4, acting as SA receptors with differential NPR1 binding affinities, were identified as adapter proteins for the CUL3-E3 ligase complex, promoting NPR1 degradation at different SA concentrations. Under normal conditions, NPR1–NPR4 interaction constantly removes NPR1 protein. Therefore, a basal level of SA is required to disrupt the NPR1–NPR4 protein interaction and maintain the basal NPR1 protein level in the cell [26].

Recent studies indicate significant crosstalk between SA and ABA during abiotic stress responses [3,27]. During a pathogen attack, the phytohormone ABA positively regulates the immune response downstream of SA by closing stomata, which provide a passive pathogen entrance passage [28,29]. Notably, in most cases, ABA levels markedly increase after SA induction during the plant–pathogen interplay [30,31]. However, if a pathogen successfully invades the apoplast, ABA acts as a negative regulator of the immune response by repressing SA biosynthesis, immune signaling, and resistance protein activity [6,32,33].

NPR1 reportedly regulates the expression of pathogenesis-related (PR) genes by binding to TGA transcription factors [34,35]. Moreover, WRKY family transcription factors are transcriptionally induced during pathogen infections, and under cold, drought, and high salinity conditions [36,37]. Genes induced during biotic and abiotic stresses often contain WRKY transcription factor binding sites (W-boxes) in their promoter regions [38]. For instance, SA biosynthesis genes contain W-boxes in their promoter regions, and *NPR1* itself is under the transcriptional control of *WKRY* genes [19,38]. Eight *WRKY* genes have been identified as direct NPR1 targets during biotic and abiotic stress responses [39]. Among proteins, WRKY18, WRKY40, and WRKY60 physically interact with each other through leucine-zipper motifs at their N-termini [40]. *WRKY18* overexpression enhances *PR* expression and resistance to invading pathogens [41]. Interestingly, WRKY18 and WRKY60 are also shown to positively regulate ABA signaling [42]. However, the exact mechanism by which NPR1 affects ABA signaling is yet to be identified.

Here, we report that NPR1 positively regulates ABA signaling via *WRKY18* transcriptional activation. We observed that loss-of-function *npr1-1* mutant seedlings were ABA insensitive during germination and cotyledon greening. Furthermore, compared to wild-type (Col-0), ABA-responsive genes were downregulated in *npr1-1* plants. More interestingly, HOS15, a substrate receptor for the DDB1-CUL4 E3 ligase complex, interacted with and regulated NPR1 stability in an ABA-dependent manner. Taken together, our findings reveal a molecular mechanism underlying the posttranslational modification of NPR1 through HOS15 and highlight the critical role of NPR1 in regulating ABA signaling.

## 2. Materials and Methods

### 2.1. Plant Materials and Growth Condition

In the present study, *Arabidopsis thaliana* ecotype Columbia (Col-0) was used as the wild-type (WT). Seeds of *hos15-2*, *npr1-1* mutants, *35S::NPR1-GFP/npr1-1/hos15-2* and *3535S::NPR1-GFP/npr1-1* were reported by Shen et al. (2020) [43]. Seeds of mutant *abi2-2* (SALK_015166C) were obtained from ABRC. Seeds were surface-sterilized and germinated on 1/2 MS medium supplemented with 0.25% phytagel (horizontal MS plates) or 1.2% agar (vertical MS plates) and 2% sucrose. Plants were grown at 23 °C under long-day conditions (16 h light/8 h dark photoperiod; energy fluence rate of 80–100 µmol m^−2^ s^−1^) in a controlled culture room.

### 2.2. Gene Expression Analysis

We used 2 µg of total RNA extracted from seedlings using the RNeasy Plant Mini Kit (Qiagen, MD, USA), treated with DNase (SIGMA Chemical Co., St. Louis, MO, USA), for the synthesis of the first-strand cDNA using the Thermoscript^TM^ RT-PCR System (Invitrogen, Paisley, UK). We performed PCR amplification using e-Taq DNA polymerase (Solgent, Daejeon, Korea). The primers used in the RT-PCR or real-time PCR experiments are listed in Supplementary Table S1. The conditions of real-time PCR were as follows: 95 °C for 5 min, 45 cycles of 95 °C for 10 s and 60 °C for 30 s, followed by 95 °C for 10 s, 65 °C for 5 s, and 95 °C for 5 s.

### 2.3. Immunoblot Analysis and Immunoprecipitation

We used 10-day-old *Arabidopsis* plants, either treated or untreated with ABA or MG132, for the Western blot assays. Total proteins were extracted and immunoblot analysis was carried out using α-GFP and α-HOS15, for GFP-tagged line and HOS15 detection, respectively. Each immunoblot was incubated with the appropriate primary antibody (α-HOS15 antibody, 1:5000; α-GFP antibody, 1:3000) for 2 h at room temperature or overnight at 4 °C. The membranes were developed using a peroxidase-conjugated secondary antibody for α-rabbit antibody (GE, Little Chalfont, Buckinghamshire, UK) at a dilution of 1:4000. We carried out the co-immunoprecipitation studies in the tobacco transient assay by co-infiltration of tobacco (*Nicotiana benthamiana*) leaves with *HOS15-GFP* and *NPR1-HA*. The protein samples were extracted three days after the infiltration. For the co-immunoprecipitation studies, we extracted the total protein samples from *N. benthamiana* leaves and performed the pull-down assay using α-GFP, followed by immunoblotting with α-HA. Each immunoblot was incubated with the appropriate primary antibody, α-HA or α-GFP antibody, diluted at 1:2000 or 1:2000, for 2 h at room temperature or overnight at 4 °C, respectively. The membranes were developed using α-rat IgG (Sigma, St. Louis, MO, USA) and α-rabbit (GE, Little Chalfont, Buckinghamshire, UK) peroxidase-conjugated secondary antibodies, diluted at 1:1000 and 1:2000, respectively.

### 2.4. Yeast Two-Hybrid Analysis

The full-length *NPR1* and *HOS15* coding sequences were cloned into the gateway entry vector, *pDONR^TM^/Zeo* and then sub-cloned into the destination vectors, pDEST22 or pDEST32, containing activation and binding domains, respectively. The primers used for cloning are listed in Table S1. The plasmids were transformed into the yeast strain PJ694A. Three independent transformants were tested for the interactions. Empty vectors were used as negative controls.

## 3. Results

### 3.1. NRP1-1 Mutant Shows ABA Insensitive Phenotype

**Figure 1:**
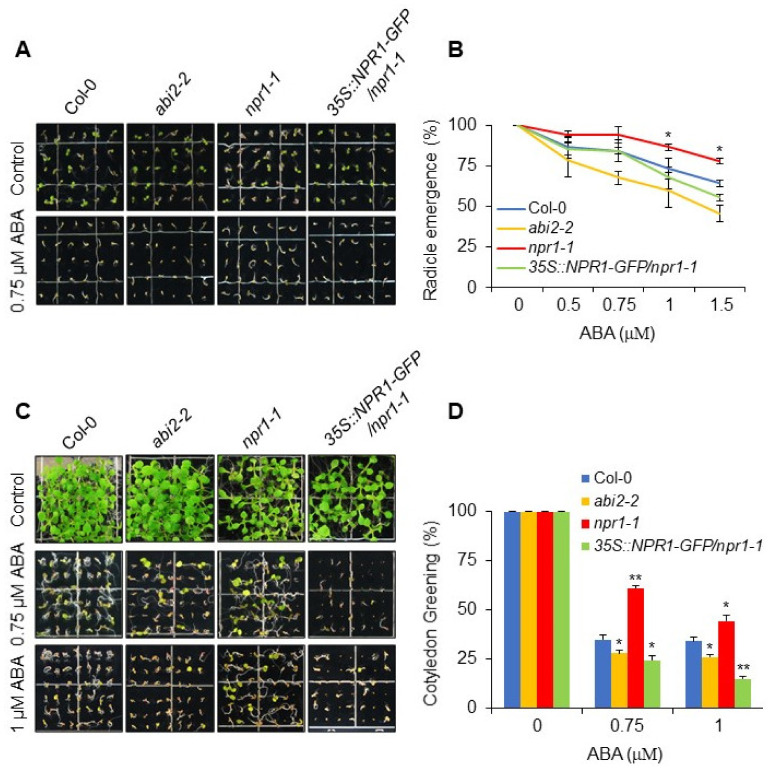
*npr1-1* mutant exhibits an ABA-insensitive phenotype. (**A**) *npr1-1* plants show ABA insensitivity. Seeds of Col-0, *abi2-2*, *npr1-1*, and *35S::NPR1-GFP/npr1-1* grown on ½ MS media with indicated concentration of ABA. Photographs were taken 5 days after germination. (**B**) Radicle emergence of indicated genotypes grown on ½ MS media with ABA. Error bars represent SE. Significant difference was determined by a student’s t-test with a *p*-value < 0.05 (*). (**C**) ABA insensitivity of *npr1-1* plants becomes more evident as the treatment time was extended to 2 weeks. Seeds of Col-0, *abi2-2*, *npr1-1*, and *35S::NPR1-GFP/npr1-1* were germinated on 1/2 MS medium supplemented with 0, 0.75 μM and 1 μM ABA. Photographs were acquired 2 weeks after germination. (**D**) The number of green cotyledons from each line was counted after 10 days of treatment with 0.75 μM and 1 μM ABA. The error bars represent the standard error (SE; *n* = 3, independent experiments performed in triplicate). Significant difference was determined by a student’s *t*-test with a *p*-value of <0.05 (*) or <0.01 (**).

Mutant *npr1-1* was isolated as a non-expresser of *PR* gene 1 in SA signaling, lacking the expression of *PR1*, *PR5*, and *BGL2* genes in response to SA, 2,6-dichloroisonicotinic acid (INA), and avirulent bacterial pathogens [44]. To determine the possible role of NPR1 in abiotic stresses, we tested if NPR1 affected ABA signaling. Seeds of wild-type (Col-0), *npr1-1*, and *35S::NPR1-GFP*/*npr1-1* plants, along with the ABA-hypersensitive *abi2-2* mutant as experimental control for the ABA response, were grown in the presence of exogenous ABA for one week. The *npr1-1* mutant displayed an ABA-insensitive phenotype during germination (Figure 1A). The *35S::NPR1-GFP*/*npr1-1* plants showed sensitivity to ABA that was intermediate between the wild-type and *abi2-2* controls (Figure 1A). The emerging radicles were counted, which showed that *npr1-1* germination resulted in the highest number of radicles among all tested lines (Figure 1B). As shown in Figure 1C,D, the ABA-insensitivity was maintained in the *npr1-1* plants after an additional growth period of 2 weeks, while Col-0 and, in a marginal way, *abi2-2*, started greening. The *35S::NPR1-GFP*/*npr1-1* plants were still hypersensitive and showed fewer green cotyledons (Figure 1C,D). Taken together, these results suggest that NPR1 positively regulates ABA signaling.

### 3.2. NPR1 Positively Regulates the Expression of ABA-Responsive Genes

**Figure 2:**
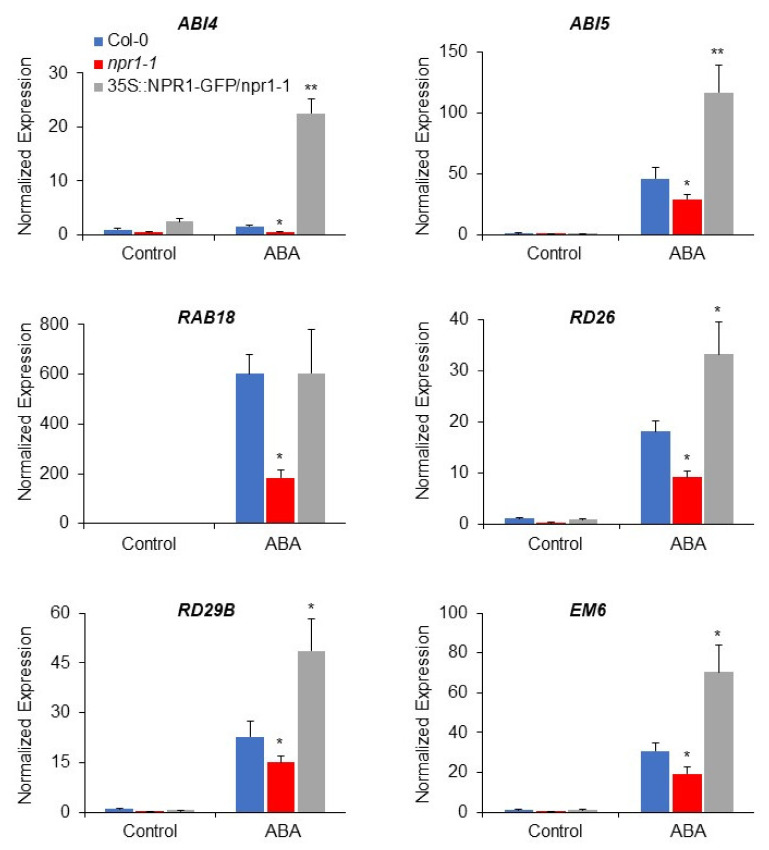
Expression analysis of downstream ABA-responsive genes in *npr1-1* and *35S:NPR1-GFP*/*npr1-1.* Relative mRNA level of ABA-responsive genes in the presence and absence of ABA in Col-0, *npr1-1*, and *35S::NPR1-GFP*/*npr1-1* determined by qRT-PCR using total RNA extracted from 7-day-old seedlings treated without (Control) or with 100 µM ABA for 6 h in MS liquid media. Ubiquitin was used as a control for normalization. The bars represent the mean ± standard error of three biological samples with technical replicates. Significant difference was determined by a student’s *t*-test with a *p*-value of <0.05 (*) or <0.01 (**).

Since *npr1-1* showed ABA insensitivity both at the early stage and during the prolonged treatment period (Figure 1), we aimed at investigating whether NPR1 could also regulate downstream ABA-responsive genes, as NPR1 has already been shown to function as a transcriptional coactivator [45]. To test our hypothesis, 7-day-old Col-0, *npr1-1*, and *35S::NPR1-GFP*/*npr1-1* seedlings were treated with 100 µM ABA, and the transcript abundance of *ABI4, ABI5, EM6, RAB18, RD26*, and *RD29B* was analyzed. The expression of all the above-mentioned genes was downregulated in the *npr1-1* mutant and upregulated in the *35S::NPR1-GFP*/*npr1-1* plants compared to the wild-type (Col-0) control (Figure 2), except for *RAB18*, which was downregulated in the *npr1-1* mutant but showed no difference between Col-0 and *35S::NPR1-GFP*/*npr1-1* plants.

To inspect the role of NPR1 more comprehensively, we directly germinated Col-0 and *npr1-1* seeds on ABA-containing MS plates and allowed them to grow for 7 days. The transcript levels of ABA-responsive genes *ABI5, EM6, RAB18, RD26*, and *RD29B* were less upregulated by ABA in *npr1-1* mutant than in Col-0 plants (Figure S1). These results demonstrated that the loss of NPR1 function leads to ABA signaling suppression, resulting in ABA-insensitive phenotypes.

### 3.3. HOS15 Interacts with NPR1 and Promotes Its Degradation

**Figure 3:**
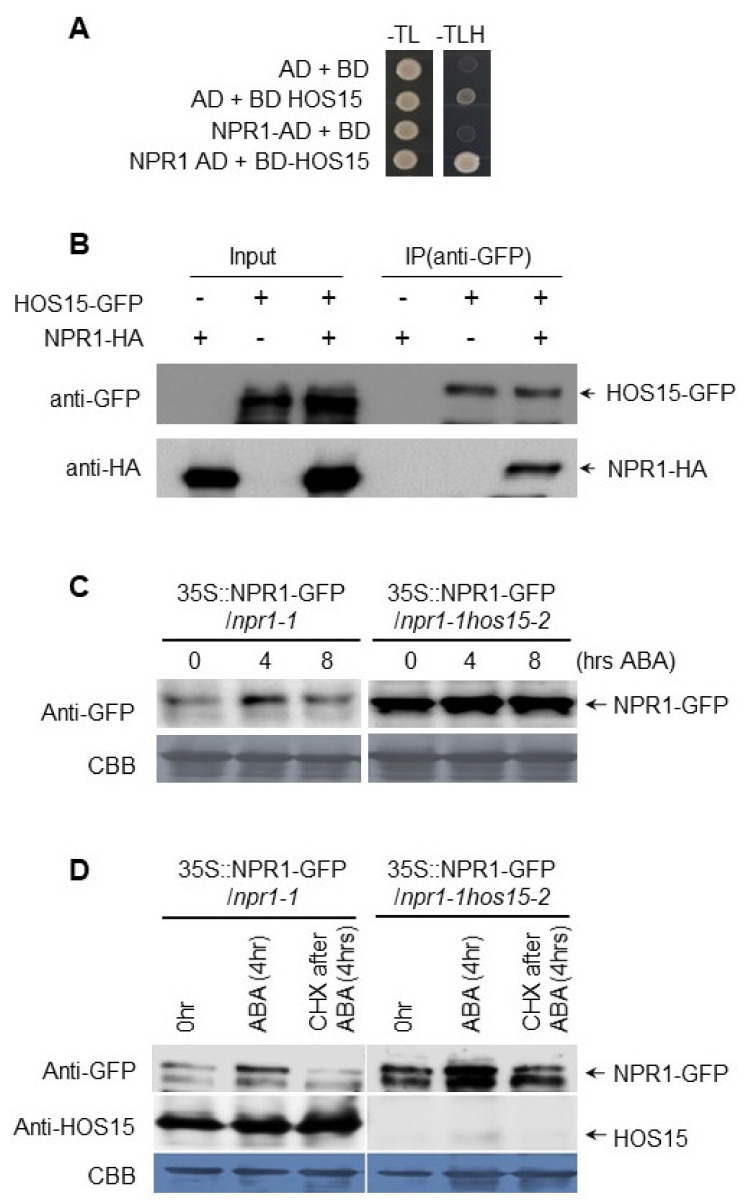
HOS15 interacts with and regulates NPR1 protein abundance. (**A**) HOS15 interacts with NPR1 in yeast. The co-transformed yeast strains were plated onto the control −TL selective −TLH media. The combinations with the empty plasmids were used as negative controls. BD, *pDEST32* (bait plasmid); AD, *pDEST22* (prey plasmid). (**B**) Co-immunoprecipitation assay between HOS15 and NPR1. Protein extracts obtained from *N. benthamiana* leaves infiltrated with *Agrobacterium tumefaciens* harboring *35S::HOS15-GFP* and *35S::NPR1-HA* were analyzed using anti-GFP and anti-HA antibodies, respectively. The protein extracts (input) were immunoprecipitated using anti-GFP antibody. The immunoblots were analyzed using anti-GFP and anti-HA antibodies to detect the interaction between HOS15 and NPR1. (**C**) The NPR1 protein degradation was determined using 10-day-old seedlings of *35S::NPRI-GFP*/*npr1-1* and *35S::NPRI-GFP*/*npr1-1 hos15-2* treated with 100 μM ABA. The samples were collected at the indicated time point, and NPR1 protein level was measured using anti-GFP antibody. (**D**) Ten-day-old seedlings of *35S::NPRI-GFP*/*npr1-1* and *35S::NPRI-GFP*/*npr1-1 hos15-2* were pretreated with 100 μM ABA for 4 h and then ABA was washed out, and the seedlings were retreated with 200 μM cycloheximide (CHX) for the next 4 h. Samples were collected and analyzed with Western blotting using anti-GFP and anti-HOS15 antibodies.

Recently, we have shown that HOS15, a WD40-repeat-containing protein, negatively regulates ABA signaling through OST1/SNRK2.6 degradation [46]. HOS15 reportedly functions as a substrate receptor for the DDB1-CUL4 E3 ligase complex [43,47,48]. Furthermore, we described that HOS15 interacts with and regulates NPR1 protein abundance, thereby negatively regulating *PR1* expression [43]. To test whether HOS15 regulates NPR1 protein abundance in response to ABA, we first confirmed HOS15 and NPR1 interaction using yeast two-hybrid and co-immunoprecipitation assays (Figure 3A,B). Next, we investigated NPR1 protein levels in transgenic plants of genotype *35S::NPRI-GFP/npr1-1* and *35S::NPRI-GFP/npr1-1/hos15-2*. As shown in Figure 3C, the NPR1 protein abundance slightly increased with ABA treatment after 4 h and then returned to control levels after 8 h. In contrast, NPR1 was highly accumulated in *hos15-2* knockout plants in an ABA-independent manner (Figure 3C). Next, we tested NPR1 levels by treating *35S::NPRI-GFP/npr1-1* and *35S::NPRI-GFP/npr1-1/hos15-2* seedlings with ABA for 4 h, subsequently washed ABA, and treated them with cycloheximide (CHX) for 4 h to inhibit de novo protein synthesis and investigate NPR1 protein stability. When treated with CHX, the NPR1 abundance declined very rapidly in *35S::NPRI-GFP/npr1-1* plants (Figure 3D). However, the NPR1 protein level remained more abundant in the *hos15-2* background compared to that in the *35S::NPRI-GFP*/*npr1-1* plants (Figure 3D), indicating that HOS15 promotes NPR1 degradation. It must be noted that the NPR1 protein levels also dropped in *hos15-2* plants after 4 h of CHX, suggesting the action of other E3 ligases that regulate NPR1 level]. Ubiquitination of NPR1 by CUL3 ^NPR3/NPR4^ E3 ligase complex has previously been reported [49]. To test whether HOS15 also regulate NPR1 level through ubiquitination, we performed ubiquitination assay. As shown in Figure S2, NPR1 was highly ubiquitinated in WT; however, we found that in the presence of ABA the ubiquitination of NPR1 was partially reduced (Figure S2). In contrast, the ubiquitination of NPR1 was partially reduced in *hos15-2* plants, suggesting the involvement of HOS15 in the ubiquitination of NPR1 (Figure S2).

### 3.4. NPR1 Functions Downstream of HOS15 in Response to ABA

**Figure 4:**
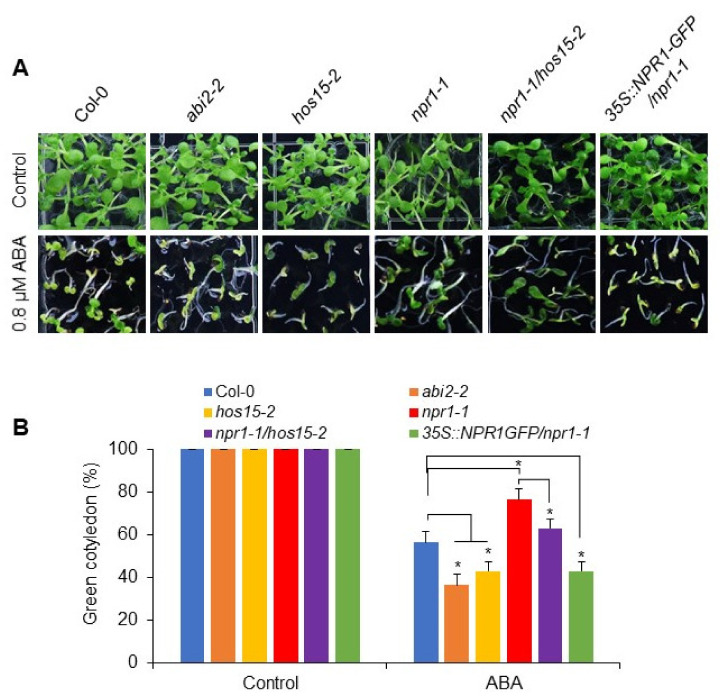
*npr1-1* mutation is epistatic to *hos15-2*. (**A**) *npr1-1* mutation suppresses the ABA sensitive phenotype of *hos15-2*. Seeds of Col-0, *abi2-2*, *hos15-2*, *npr1-1*, *npr1-1/hos15-2* double mutant, and 35S::NPR1-GFP/*npr1-1* strains were germinated on 1/2 MS medium with the indicated supplement of ABA. Photographs were acquired 2 weeks after germination. (**B**) The green cotyledons in (**A**) were counted 2 weeks later, with error bars representing the SE (n = 3 independent experiments performed in triplicate). Significant difference was determined by a student’s *t*-test with a *p*-value < 0.05 (*).

As described in Figure 1, *npr1-1* plants show ABA-insensitivity, whereas *hos15-2* plants reportedly exhibit an ABA-sensitive phenotype [46]. Therefore, we aimed at investigating the epistatic effects of these two mutations. We thus crossed *npr1-1* plants with *hos15-2* plants and germinated their seeds on ABA-containing MS media. As expected, the *npr1-1* plants were insensitive, whereas the *hos15-2* plants were sensitive to ABA (Figure 4A,B). Interestingly, the *npr1-1/hos15-2* double mutant plants were less sensitive to ABA than the *hos15-2* single mutant (Figure 4A,B). Moreover, *NPR1*-overexpressing plants showed ABA hypersensitivity and a lower quantity of green cotyledons than the wild-type plants (Figure 4A,B). Taken together, these results demonstrate that NPR1, which functions downstream of HOS15, positively regulates the ABA-mediated seed germination.

### 3.5. NPR1 Regulates the Transcription of WRKY Genes

**Figure 5:**
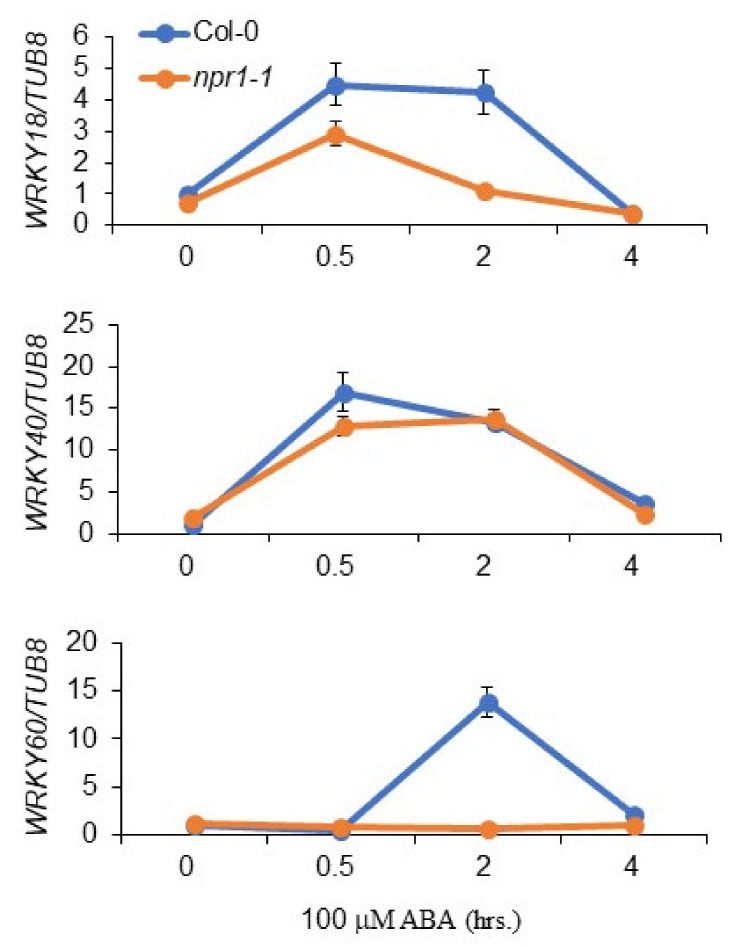
NPR1 regulates *WRKY* gene expression in an ABA-dependent manner. The abundance of *WRKY* transcripts in *npr1-1* plants under ABA stress defines their ABA-insensitive phenotype. The expression of *WRKY* genes in Col-0 and *npr1-1* strains. Seeds of wild-type (Col-0) and *npr1-1* plants were cultured on 1/2 MS medium for 2 weeks and then treated with 100 μM ABA for the indicated durations (0, 30 min, 2 h, and 4 h). Total RNA was extracted from the seedlings and analyzed using RT-qPCR. *TUB8* was used as an internal control. The error bars indicate SD.

Previous studies have shown that WRKY18 positively regulates ABA signaling by activating the transcription of *WRKY60* through competition with WRKY40, a reported negative regulator of ABA signaling [42]. It has also been reported that NPR1 is directly associated with the *WRKY18* gene promoter [39]. Therefore, we next investigated the transcript abundance of these *WRKY* genes in *npr1-1* plants. As expected, upon treatment with ABA, *WRKY18* expression was downregulated in *npr1-1* plants compared to wild-type plants (Figure 5). *WRKY60*, a direct target of WRKY18, was also downregulated in *npr1-1* plants (Figure 5). In contrast, we could not find any change in the transcriptional abundance of *WRKY40* (Figure 5). Taken together, these results demonstrate that NPR1 is involved in the ABA signaling regulation through the positive regulation of *WRKY18* transcription. However, we could not completely exclude further possible roles of NPR1 in ABA biosynthesis and/or signaling at this stage.

## 4. Discussion

### 4.1. NPR1 Positively Regulates ABA Response

The *Arabidopsis npr1-1* mutant has been isolated as a carrier strain of a recessive point mutation, which abolished SAR-responsive PR gene expression [44]. Plants carrying the *npr1-1* point mutation lacked the expression of SA-, INA- (synthetic SA-analog) and chimeric pathogen-responsive reporter genes. Earlier reports have also shown that the *npr1-1* point mutation results in SA-, INA- and pathogen-insensitive phenotypes during SAR induction, indicating that these inducers potentially share common signaling pathways [44]. However, the role and effector mechanism of NPR1 function in other signaling pathways, such as the ABA signaling cascade, remain elusive. We observed that the *npr1-1* mutant showed an ABA-insensitive phenotype during early germination and post-germination seedling growth (Figure 1). Furthermore, ABA-responsive gene expression was also downregulated in the *npr1-1* mutant, while it was upregulated in the overexpressing line compared with the wild-type (Figure 2). Taken together, these results indicate that NPR1 positively regulates the ABA signaling pathway.

### 4.2. NPR1 Is a Target of the CUL4-DDB1-HOS15 E3 Ubiquitin Ligase Complex

Previous reports have suggested that *Arabidopsis* HOS15 functions as a repressor protein in abiotic stress-related gene expression regulation through chromatin modification [47]. We have recently shown that HOS15 contains a WD40-repeat domain that functions as a substrate receptor for the CUL4-DDB1 E3 ligase complex [43,47]. CUL4 itself is reportedly a negative regulator of ABA signaling, and it is involved in the proteasomal degradation of ABI5, involving ABD1, DWA1, and DWA2 (WD40 repeat proteins) (summarized by Ali et al., 2020) [50], and the *cul4cs* mutant line displays an ABA-sensitive phenotype [51]. As shown in Figure 3, NPR1 interacts with HOS15, a part of the CUL4-DDB1 complex, suggesting that NPR1 is a target of the CUL4-DDB1 E3 ligase complex that may be involved in NPR1 protein degradation. Ubiquitination is a common mechanism to promote target protein degradation. Plant growth and development are largely affected by the ubiquitin-mediated degradation of target protein stability, whereas the recognition and target specificity of the ubiquitination pathway is mainly controlled by the substrate recruitment of E3 ubiquitin ligases [52,53]. Previous studies have shown that NPR1 is degraded by CUL3-E3 ligase in the presence of ABA [49]. However, NPR1 degradation was not completely blocked in the absence of CUL3, raising the possibility that CUL3 might be not the only E3 ligase that promotes NPR1 degradation. Therefore, we investigated the CUL4-DDB1 complex-dependent NPR1 protein stability through HOS15, as NPR1 interacts with this complex (Figure 3A,B) [43]. The NPR1 protein level slightly increased by the 4-h ABA treatment, then quickly reduced after 8 h in WT plants (Figure 3C). In contrast, NPR1 was continuously stable in *hos15-2* knockout plants, suggesting that HOS15 promotes NPR1 degradation (Figure 3C). During the cycloheximide (CHX)-mediated blocking of the protein synthesis, the NPR1 protein was degraded markedly faster in WT compared to *hos15-2* (Figure 3D). These results strongly suggest that the CUL4-DDB1-HOS15 ubiquitin E3-ligase complex mediates NPR1 proteasomal degradation.

### 4.3. NPR1 Acts Downstream of HOS15 and Regulates WRKY Gene Expression

The *cul4cs* mutants reportedly display a hypersensitive phenotype to exogenously applied ABA and negatively regulate the ABA signaling pathway [51]. Interestingly, the *hos15-2* loss-of-function mutant, lacking the substrate receptor for the CUL4-DDB1 E3 ligase complex, also shows an ABA-hypersensitive phenotype [47], indicating that HOS15 and CUL4 function together as negative regulators of ABA signaling. Furthermore, we recently reported that NPR1 is a target of the CUL4-DDB1-HOS15 E3 ligase complex in the context of NPR1-mediated activation of plant immunity [43]. Since *npr1-1* shows ABA insensitivity, while *hos15-2* shows an ABA-sensitive phenotype, we tested the phenotype of the *npr1-1/hos15-2* double mutant plants (Figure 1). Interestingly, the NPR1 mutation (*npr1-1*) suppressed the ABA sensitivity of *hos15-2* in the double mutant (Figure 4), indicating that NPR1 acts downstream of HOS15 in ABA signaling.

**Figure 6:**
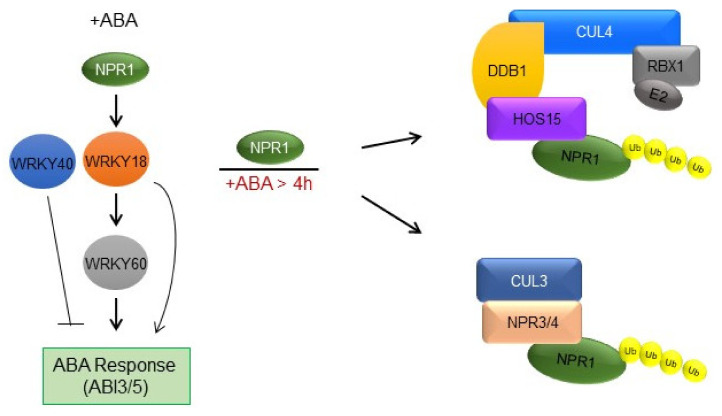
Hypothetical model. During ABA stress, NPR1 regulates ABA-responsive stress-related gene expression and activates *WRKY18*, which in turn competes with *WRKY40* to regulate *WRKY60* and activate the downstream ABA response. However, NPR1 interacts with HOS15, a substrate receptor for the CUL4-DDB1 E3-ligase ubiquitin complex, and gradually, after the activation of the WRKY genes and ABA response, NPR1 is degraded in an ABA-dependent manner. NPR1 is also degraded by CUL3 ^NPR3/NPR4^ E3 ligase complex in an ABA dependent manner [Modified from, [49]].

NPR1 can directly associate with various *WRKY* gene promoters [39]. Among these, WRKY18, which is the direct target of NPR1, has been shown to interact with WRKY40 through a leucine-zipper motif at their N-terminal regions [40]. WRKY18 overexpression results in the hyper-induction of the *PR* gene and confers resistance to invading pathogens [41]. Interestingly, WRKY18 and WRKY60 are also reportedly involved in the positive regulation of ABA signaling and the regulation of ABA-responsive genes by directly regulating ABI4 and ABI5 promoters [42]. The expression of *WRKY18* was downregulated in *npr1-1*, whereas that of *WRKY60* was not induced at all in *npr1-1* in response to ABA (Figure 6). These results indicate that NPR1 activates WRKY18 expression in the very early stage of ABA stress, which in turn activates WRKY60 to regulate the ABA response.

In addition to dehydration responses, plants also close their stomatal pores as part of their innate immunological response to keep bacteria out [28,29]. The role of NPR1 as one of the major regulators of plant immunity has already been well documented [21,22,23,43,45]. The identification of NPR1 as a positive regulator of ABA signaling now highlights its involvement in ABA-dependent pathogen response, which represents a major goal for future studies.

### 4.4. Working Model for the Role of NPR1 in the ABA Pathway

ABA is a major phytohormone that plays a crucial role in biotic and abiotic stress responses [3,4,54]. The ABA signaling pathway is well characterized and recent research efforts have focused primarily on exploring more regulatory components of this widely studied signaling cascade. Our findings suggest that NPR1 positively regulates ABA signaling by activating the transcription of *WRKY18*, which regulates *WRKY60* and the ABA response (Figure 6). However, NPR1 interacts with HOS15, a substrate receptor for the CUL4-DDB1 E3-ligase complex, which promotes NPR1 degradation upon ABA.

## Data Availability

All the data which is presented in this paper are available in manuscript main text and Supplementary Materials.

## Supplementary Materials

The following supporting information can be downloaded at: https://www.mdpi.com/article/10.3390/plants11060815/s1, Figure S1: Expression analysis of downstream ABA-responsive genes in Col-0 and *npr1-1*; Figure S2: HOS15 promotes NPR1 ubiquitination. NPR1 is ubiquitinated in by HOS15; Table S1: List of qRT-PCR primers.
