# Supplementary material for: Non-Expresser of PR-Genes 1 Positively Regulates Abscisic Acid Signaling in Arabidopsis thaliana
Source: Plants (Basel). 2022 Mar 18;11(6):815. doi: 10.3390/plants11060815 (PMC8950481; doi:10.3390/plants11060815)
Supplement: Supplementary file 1 [file plants-11-00815-s001.zip › plants-1528354-supplementary.pdf]

plants MDPI

Supplementary Materials:

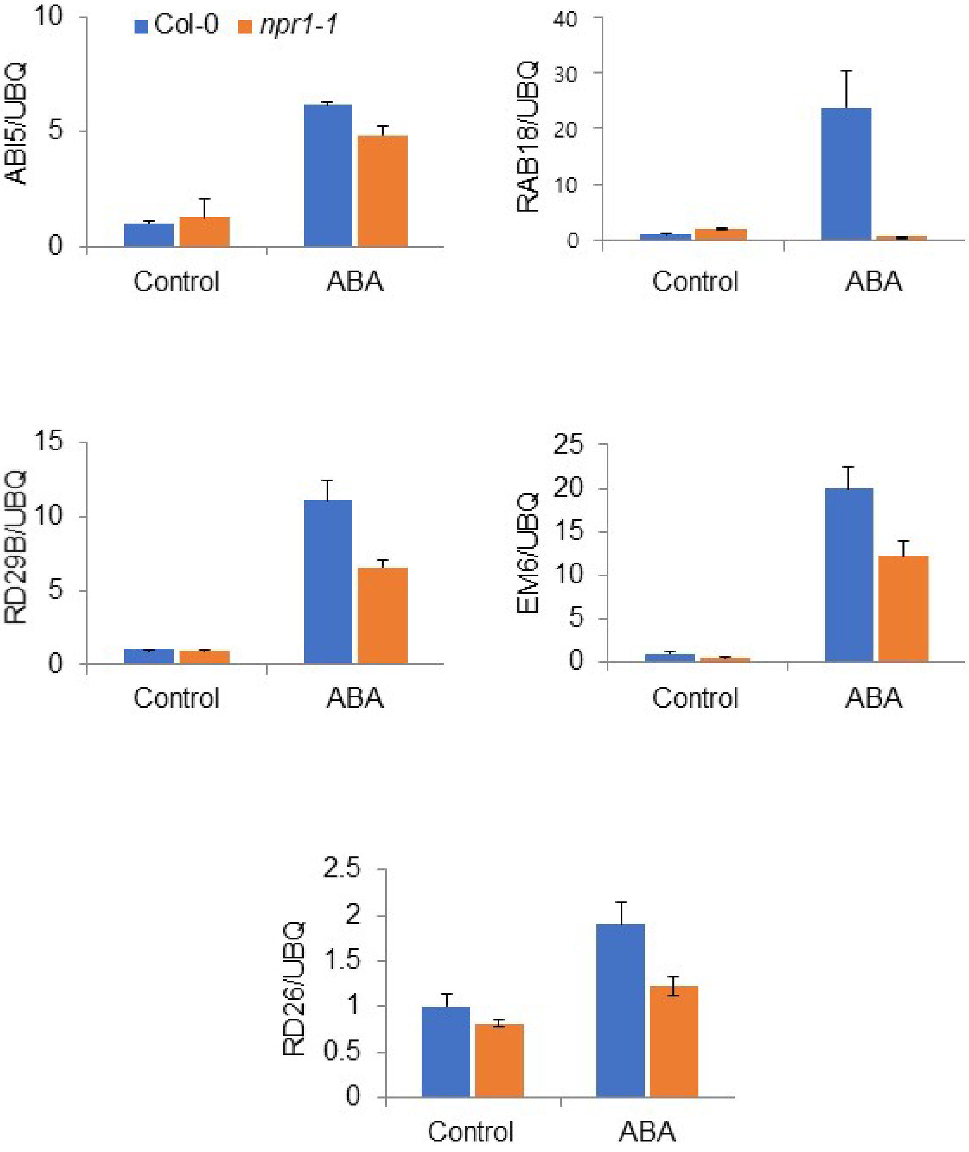

Figure S1. Expression analysis of downstream ABA-responsive genes in Col-0 and *npr1-1*. Col-0, *npr1-1*, and 35S::NPR1-GFP/*npr1-1* seeds were directly germinated on ½ MS (Control) and 0.2 μM ABA-containing media (ABA), seedlings were grown for 7 days, total RNA was extracted, and the indicated gene expression was determined by qRT-PCR analysis. *TUB8* was used as an internal control. The error bars indicate ± SD. Significant difference was determined by a Student's t-test with a *p*-value of <0.05 (*) or <0.01 (**).

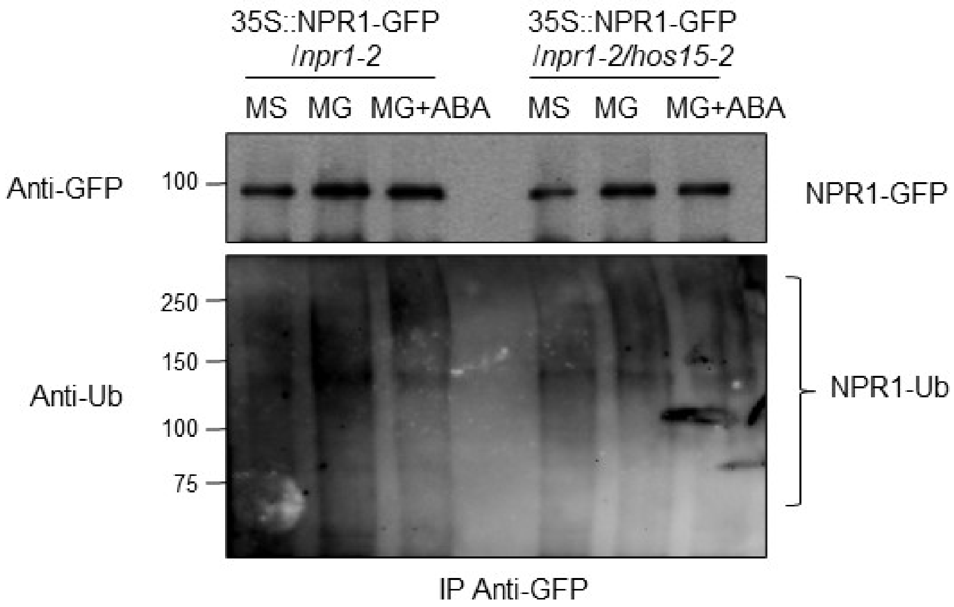

Figure S2. HOS15 promotes NPR1 ubiquitination. NPR1 is ubiquitinated in by HOS15. Ten-day-old seedlings of Col-0, *35S::NPRI-GFP/npr1-2* and *35S::NPRI-GFP/npr1-2/hos15-2* were treated without (MS) or with MG132 and ABA for 8 h. Total proteins were extracted and immunoprecipitated with anti-GFP antibody. Immuno-precipitated proteins were analyzed using anti-GFP and anti-Ubiquitin antibodies. NPR1 is highly ubiquitinated in WT (Col-0), while ubiquitination of NPR1 was reduced in *hos15-2*.

Table S1. List of qRT-PCR primers.

PRIMERS	SEQUENCE (5' to 3')	PURPOSE
attB1 adapter	GGGGACAAGTTTGTACAAAAAAGCAGGCT	Plasmid construction
attB2 adapter	GGGGACCACTTTGTACAAGAAAGCTGGGT	
HOS15 attB1	AAAAAAGCAGGCTTCATGTCTTCACTTACCTCC	
HOS15 attB2	AGAAAGCTGGGTCCATTCTGAAATCAAGAA	
HOS15 no attB2	AGAAAGCTGGGTGCATTCTGAAATCAAGAACG	
NPR1 attB1	AAAAAAGCAGGCTTCATGGACACCACCATTGATGGA	
NPR1 attB2	AGAAAGCTGGGTCTCACCGACGACGATGAGAGAG	
ABI4 QRT-F	GTCCAGATGGGACAATTCCAACACC	qRT-PCR
ABI4 QRT-R	CCCTAACGCCACCTCATGATGAAAC	
ABI5 QRT-F	AAGCCACCGGTTTTTAGACACACAG	
ABI5 QRT-R	CACCTCCTCCATTATGTCTCGCTTG	
RAB18 QRT-F	TTCGGTCGTTGTATTGTGCTT T	
RAB18 QRT-R	CCAGATGCTCATTACACACTCATG	
RD26 QRT-F	GAAGGTGAGGCGGAGAGTG	
RD26 QRT-R	CCCGAAACTCTGAGTCAACCT	
RD29B QRT-F	CTGATCCCACGCATAAAGGT	
RD29B QRT-R	TCCATCCCAGCTTTTGATTC	
EM6 QRT-F	GCAAAGAAGGGCGAGACC	
EM6 QRT-R	TCCTCCTCAGCGTGTTCC	
WRKY18 QRT-F	CGTGCCTACTGAAACATCGGAC	
WRKY18 QRT-R	GTAAGCTCTAGGTGACGGGTTGTC	
WRKY40 QRT-F	AAATCAGCCCTCCCAAGAAACG	
WRKY40 QRT-R	CTTCACGACAGTCTCTTCTCTCTGC	
WRKY60 QRT-F	GGTGGGCTTGAACCAGTTGAGG	
WRKY60 QRT-R	AATCTCCCGGAAATAGCAGTCG	
TUB8 qRT-F	CGT GGA TCA CAG CAA TAC AGA GCC	
TUB8 qRT-R	CCT CCG CAC TTC CAC TTC GTC TTC	
